# 2573. The Use of MRSA PCR for De-escalation of Anti-MRSA therapy for CAP and HAP at a Community Hospital: a Quality Improvement Analysis for Antibiotic Stewardship

**DOI:** 10.1093/ofid/ofad500.2190

**Published:** 2023-11-27

**Authors:** Angelina M Browne, Mark Rasnake, Stephanie Ducas

**Affiliations:** NCH Healthcare System, Naples, Florida; NCH Healthcare System, Naples, Florida; NCH Healthcare System, Naples, Florida

## Abstract

**Background:**

The use of nasal MRSA PCR is beneficial in reducing the duration of anti-MRSA therapy, since it can rapidly be attained and has excellent negative predictive value. There is scarcity of literature regarding the potential impact of MRSA PCR on the length of stay and cost of care at a community hospital, when considering empiric treatment of CAP or HAP.

**Methods:**

This retrospective analysis was conducted at Naples Community Hospital, a 588-bed facility. We assessed 134 patients that were admitted for treatment of pneumonia who received anti-MRSA therapy for CAP or HAP from March to April 2023. Our approach was to employ a pharmacy-driven protocol whereby adult patients who had received either vancomycin, linezolid, or both during their hospitalization were screened with MRSA nares PCR shortly after initiation of anti-MRSA therapy. The primary outcome was the duration of anti-MRSA treatment in hours. Secondary outcomes assessed included time from MRSA colonization status result to the discontinuation of anti-MRSA therapy, with subgroup analyses of the percentage of patients with vancomycin trough levels drawn in the vancomycin group, the incidence of acute kidney injury and hospital length of stay pre- and post-implementation.

**Results:**

Our analysis showed a primary outcome with a statistically significant reduction in the hours of anti-MRSA therapy duration when comparing pre- and post-implementation of MRSA PCR (64 vs. 40 hours, P= 0.0008). A considerable improvement was noted in the total duration of anti-MRSA therapy (26 vs. 11 hours, P=0.0006). Our study showed a reduced in length of stay; however, was not statistically significant between the two groups (206 vs 142 hours, P=0.066). No statistical difference was noted in the incidence of AKI between groups.

**Conclusion:**

Our study suggests that pharmacy-driven protocols employed in community hospitals can significantly aid in reduction of inappropriate use of anti-MRSA empiric therapy and reduce costs associated with unnecessary antibiotics. Our analysis showed a trend to shorter length of stay post-implementation, which shortened duration of anti-MRSA therapy. Further investigation with a larger sample size, may also show a significant reduction in length of stay.

**Disclosures:**

**All Authors**: No reported disclosures

